# Effects of ageing on responses to stepping-target displacements during walking

**DOI:** 10.1007/s00421-020-04504-4

**Published:** 2020-09-29

**Authors:** Yajie Zhang, Jeroen B. J. Smeets, Eli Brenner, Sabine Verschueren, Jacques Duysens

**Affiliations:** 1 Department of Rehabilitation Sciences, FaBer, KU Leuven, Leuven, Belgium; 2 Department of Human Movement Sciences, Amsterdam Movement Sciences, Vrije Universiteit Amsterdam, Amsterdam, The Netherlands; 3 Motor Control Laboratory, Movement Control and Neuroplasticity Research Group, FaBer, KU Leuven, Leuven, Belgium

**Keywords:** Target jump, Correction, Elderly, Leg adjustments, Force

## Abstract

**Purpose:**

Human sensory and motor systems deteriorate with age. When walking, older adults may therefore find it more difficult to adjust their steps to new visual information, especially considering that such adjustments require control of balance as well as of foot trajectory. Our study investigates the effects of ageing on lower limb responses to unpredictable target shifts.

**Methods:**

Participants walked on a treadmill with projected stepping targets that occasionally shifted in the medial or lateral direction. The shifts occurred at a random moment during the early half of the swing phase of either leg. Kinematic, kinetic and muscle activity data were collected.

**Results:**

Older adults responded later and corrected for a smaller proportion of the shift than young adults. The order in which muscle activation changed was similar in both groups, with responses of gluteus medius and semitendinosus from about 120 to 140 ms after the shift. Most muscles responded slightly later to lateral target shifts in the older adults than in the young adults, but this difference was not observed for medial target shifts. Ageing delayed the behavioural responses more than it did the electromyographic (EMG) responses.

**Conclusions:**

Our study suggests that older adults can adjust their walking to small target shifts during the swing phase, but not as well as young adults. Furthermore, muscle strength probably plays a substantial role in the changes in online adjustments during ageing.

## Introduction

When walking, the movements of one’s feet are continuously controlled based on the latest visual information (Zhang et al. 2020), which makes it possible to adjust one’s steps. Adjusting steps when walking is very common in daily life. Incorrectly adjusting a step during walking can affect balance which can lead to a fall. With ageing, the ability to maintain balance decreases, including maintaining balance during walking (Baloh et al. 2003; Schrager et al. 2008). It has been shown that the most frequent (41%) cause of falls is incorrect bodyweight shift (Robinovitch et al. 2013). The bodyweight shift is demanding during walking, especially during the double-stance phase when weight is shifted from one leg to the other. Therefore, older adults might adjust steps less during walking.

In a previous study, we successfully induced foot adjustments in young adults with small disturbances (2.5-cm target shifts) that would not be expected to compromise the ability to maintain balance (Zhang et al. 2020). The same small disturbance was used in the present study. We used the same disturbance for young and older adults to be able to compare their responses, and used small disturbances to encourage older adults to try to make adjustments. Another kind of visual perturbation that affects foot placement is ‘obstacle avoidance’, in which it is forbidden to step onto an indicated location or area, so that if the foot is heading for such a location, its trajectory must be adjusted to land elsewhere (Moraes et al. 2004; Potocanac et al. 2014; Weerdesteyn et al. 2004). We used ‘stepping stones’ (targets), in which we indicated where the foot should be placed on each step, because this allowed us to control step length and duration, as well as the magnitude of a full correction after a target shift.

In general, previous studies on making step adjustments have shown that older adults have longer response times than young adults. In a task involving ‘target shifts’ or ‘target jumps’, a target represents the location at which one is to place the foot. When such a target is displaced, the foot’s trajectory is expected to be adjusted so that the foot reaches the new location (Bank et al. 2011; Barton et al. 2019; Peper et al. 2012; Young and Hollands 2012). This is one task in which delayed responses in older adults have been observed (Young and Hollands 2012). Older adults were found to be particularly poor at making corrections for sideways stepping-target shifts when walking (Hoogkamer et al. 2015; Mazaheri et al. 2015). Another task in which older adults have been found to have later responses (and a lower accuracy) when adjusting their trajectory is when stepping from quiet stance (gait initiation) (Sun et al. 2017; Tseng et al. 2009). During obstacle avoidance in walking, it was observed that muscle activation took about 10 ms longer in older adults than in young adults (Weerdesteyn et al. 2007). A similar delay was seen in tripping reactions (Schillings et al. 2005).

It is difficult to tell why sideway adjustments were so limited in older adults in previous studies, since stepping positions were usually obtained without an analysis of kinematics, kinetics or electromyography (EMG). Therefore, in the present study, we used EMG measures in conjunction with kinetics and kinematics. According to our previous study, young adults can start such an adjustment within as little as about 123 ms for muscle activation and about 155 ms for kinematics (Zhang et al. 2020). We knew that gluteus medius (GlM), semitendinosus (ST), biceps femoris (BF), tibialis anterior (TA) and gastrocnemius medialis (GaM) from both the swing and stance leg respond quickly to medio-lateral target shifts (Zhang et al. 2020). We therefore compared the muscle activity in these same muscles in older adults with what we previously observed for young adults, to see if the older adults adopt different strategies for medio-lateral step adjustments, and to see if their muscle responses are delayed.

We anticipate that the older adults will have later adjustments than the young adults. We expect the age-related delay in leg adjustments to be similar to such delay in arm adjustments, since the leg adjustments are extremely fast, similar to arm adjustments (Zhang et al. 2020). In reaching tasks, the response latency to a target shift is 100–160 ms (Oostwoud Wijdenes et al. 2013; Smeets et al. 2016; Zhang et al. 2018b), and older adults have a delay of about 15–20 ms (Kadota and Gomi 2010; Kimura et al. 2015; Zhang et al. 2018a). This may be due to deterioration of sensory functions with age, such as poorer contrast sensitivity and slower processing speed in vision (Fiorentini et al. 1996; Habekost et al. 2013). The delay may also be related to deteriorated motor functions, in particular the ability to generate force (Holviala et al. 2014). The question arises whether such deficits are reflected in foot behaviour and EMG in step adjustments.

In summary, our study aims to examine the effects of ageing on the lower limbs’ responses to unexpected stepping-target shifts. We examine changes in kinematics, kinetics and muscle activity, in order to obtain insight into how ageing affects online adjustments in time and space. The combination of these methods is important since it allows us to answer the question whether delayed reactions in the elderly have a neural basis or are partly due to a peripheral deficit, for example, a limitation in the ability to quickly produce sufficient force. Indeed, such deficit has been widely noted in work on rapid voluntary stepping (Melzer et al. 2010), gait initiation (Patla et al. 1993; Sparto et al. 2014) and gait perturbations, including tripping (Pijnappels et al. 2005) and slipping (Tang and Woollacott 1998).

## Methods

### Participants

Twenty older adults (70.0 ± 5.2 years, nine males) and 20 young adults (24.3 ± 3.6 years, eight males; the group described in Zhang et al. 2020) participated in this study. All except two older participants were right-leg-dominant, as determined by asking them to imagine kicking a ball. They all had normal or corrected-to-normal vision and had no disease that is known to affect motor or sensory function. An example video was shown before walking, and they were all able to detect the target shifts and understood the task. The study was approved by the Research Ethics Committee of KU Leuven (B322201732964), and was conducted in accordance with the standards set out in the Declaration of Helsinki, registered in the local clinical trial centre (clinical trial number at UZ Leuven: S60160). Written informed consent was obtained from each participant.

### Experimental setup and procedure

**Fig. 1:**
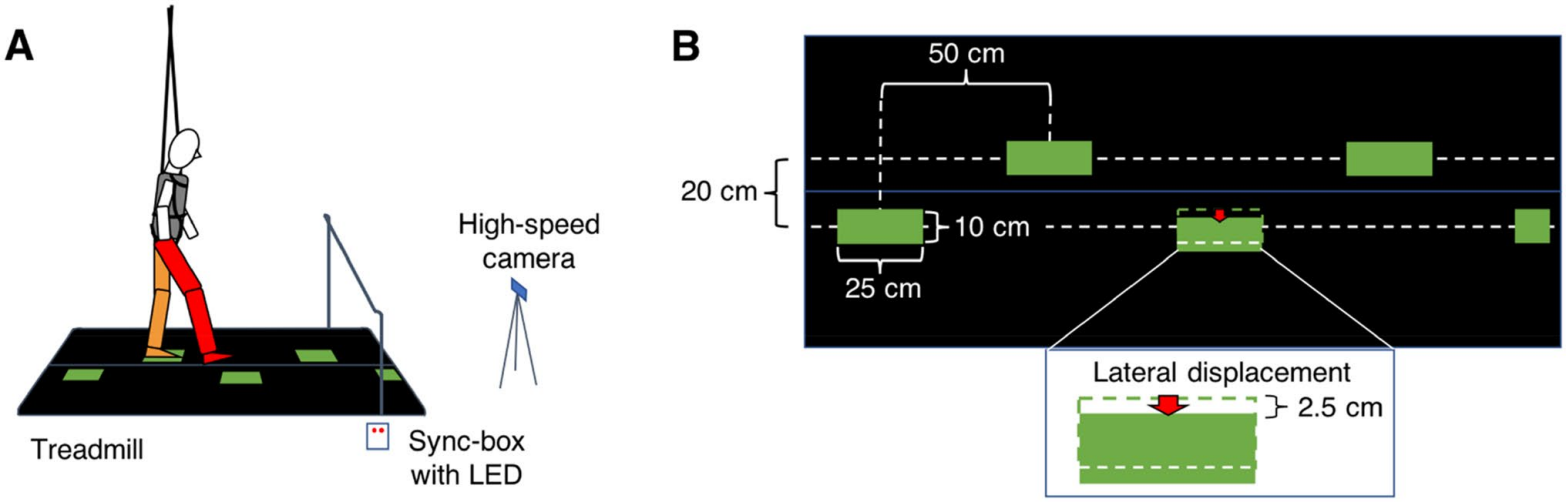
Set-up. **a** Side view of a participant who is standing on the left leg (orange). The right leg (red) is swinging to a green stepping-target that shifts laterally. The belts of the treadmill are black and cover similarly sized force plates. For clarity, the projector and the motion capture cameras surrounding the measurement field are omitted from this picture. The high-speed camera was used to determine the exact timing of the perturbation relative to the gait. **b** Top view of the treadmill with the same stimulus as in panel A. The red arrow indicates a 2.5-cm lateral displacement of a stepping target for the right leg. This figure is reproduced from Zhang et al. (2020)

The setup (Fig. 1) and procedure were the same as we used in our previous study (Zhang et al. 2020) except for the addition of a test of functional mobility, the Timed Up and Go (TUG) test (Podsiadlo and Richardson 1991; Shumway-Cook et al. 2000). For this test, participants stood up from a chair, walked three metres, turned around, walked back and sat down again at a normal pace. Before starting walking on the treadmill, the TUG test was measured twice, and the shorter time of performance was used. The data reported here for the older participants are new. For the comparison, we report the data of young adults from Zhang et al. (2020).

All participants walked at 3 km/h on an instrumented treadmill (M-Gait, Motekforce Link, the Netherlands). We chose this speed to ensure that the task would not be too difficult for the elderly (Hegeman et al. 2012; Mazaheri et al. 2015; Potocanac et al. 2015). Two 3D force plates under the spilt-belt treadmill measured the ground contact forces at 1000 Hz (Fig. 1a). A projector (Hitachi CP-AW312WN LCD, Japan) projected stepping-targets on the treadmill from the right side of the participant at an angle of about 45°. We instructed the participants to step on the stepping-targets. A safety harness prevented the participant from falling in the case of balance loss (which never occurred). Software (CueFors, Motekforce Link, the Netherlands) triggered target displacements based on the gait pattern (as described below). We recorded images of the stimulus and the participant with a high-speed video camera (Casio ER-ZR 1000, Japan; sampling rate: 240 Hz) to determine the actual moment of target shift. The video was synchronized with the 3D motion caption system (Vicon, Oxford Metrics, UK, sampling rate: 200 Hz) by a box with LED lights, connected to an external trigger (sampling rate: 1000 Hz). Following Mazaheri et al. (2015), we used the midpoint between a marker on the second toe tip and a marker on the calcaneal tuberosity to calculate the foot kinematics.

We recorded electromyographic (EMG) activity using a wireless system (Cometa Systems, Italy) at a sampling rate of 1000 Hz. We measured muscle activity from eight muscles of each leg: gluteus medius (GlM), vastus lateralis (VL), vastus medialis (VM), biceps femoris (BF), semitendinosus (ST), tibialis anterior (TA), gastrocnemius lateralis (GaL), and gastrocnemius medialis (GaM). We report EMGs of the swing leg (ipsilateral) and the stance leg (contralateral) separately. Electrodes were attached at positions following the recommendations of SENIAM (https://www.seniam.org/). Maximal voluntary contraction (MVC) was taken from the maximum of three trials of maximal isometric contraction for each muscle of each individual. This was measured before or after the treadmill experiment.

The properties of stepping-targets and their order of appearance were coded in MATLAB (The MathWorks Inc, USA) and loaded to CueFors. The stepping targets were green rectangles (25 × 10 cm). They were 50 cm apart in the direction of walking (corresponding to a swing duration of about 400 ms) and 20 cm apart laterally (Fig. 1b). Medio-lateral target shifts were always 2.5 cm. These shifts were chosen to be small enough to avoid destabilisation and to prevent crossing steps. The target shift was initiated when the participant’s centre of pressure was 65 cm from the stepping target’s position. This threshold of 65 cm corresponds to a moment early in the swing phase and was considered to be a relatively easy time at which to initiate a response in previous studies (Hoogkamer et al. 2015, 2017; Mazaheri et al. 2015). As a result of delays in the equipment and of variations in gait, the timing of the perturbations varied within a range of about 200 ms with respect to the actual foot placement. This made it necessary to get the actual frames in which the position changed from the high-speed video.

There were 10 walking episodes, each containing about 165 stepping-targets (around 2 min of walking). There were 6 perturbations for each direction (medial or lateral) for each leg (left or right) within a walking episode. These 24 perturbations were presented in a random order. The first 10 targets of each walking episode were always unperturbed. After that, a target with a perturbation occurred every five to eight steps. As each participant performed 10 walking episodes, they had to deal with 240 perturbed targets (60 per combination of direction and leg) and around 1420 unperturbed targets (around 710 per leg).

Participants first walked normally at 3 km/h without stepping-targets on the treadmill for one minute. After that, they were asked to step on a series of 120 unperturbed stepping-targets when walking on the treadmill to practice placing their feet at indicated positions. They then performed the 10 walking episodes. They rested between episodes. They knew that the perturbations would be in the medio-lateral direction, but did not know which step or which leg would be perturbed. They were instructed to step on the projected stepping targets as accurately as possible.

### Data analysis

The data analysis was largely the same as in Zhang et al. (2020). We analysed one additional dependent variable: the step angle. This is a measure of the change in orientation of the foot in response to the target shift. In comparison with the previous study, we used a slightly different rule for excluding steps when calculating the latency in each individual step (see the last part of section “Dependent variables”). Therefore, some of the results for the young participants differ slightly from the ones reported in the previous study.

- Reference with no perturbation
  The leg swing varied slightly from step to step. In addition, there was variability in the timing of the perturbations. We therefore used the large set of unperturbed steps to search for the 20 velocity profiles that best matched that of a perturbed step near the time of the perturbation (within 50 ms of the perturbation). We used the average of these 20 profiles as the reference for that perturbed step. This is more complicated than using the average of all the unperturbed steps as a reference for all perturbed steps, but it provides much clearer comparisons for individual steps (Zhang et al. 2020). We used mean values for the selected set of 20 (best matching) unperturbed steps as the reference for the corresponding perturbed step for all our analyses, including the analysis of the centre of pressure (COP) and the EMG.
- Dependent variables
  The variables describing the kinematics and centre of pressure that we report in the medio-lateral direction are signed: positive is in the same direction as the perturbation; negative is in the opposite direction. We also checked the COP in the anterior–posterior direction: positive is in the walking direction. As a measure of foot adjustment accuracy, we defined the magnitude of the correction (% of target shift) as the medio-lateral distance between the foot endpoint of a perturbed step and that of its reference (unperturbed step), divided by 2.5 cm. The midpoint between toe and heel markers at the moment of the next midstance (to ensure that the foot was flat on the treadmill) was taken as the foot’s endpoint. Swing duration was defined as the time between the toe-off moment and the heel-strike moment of the swing leg, which were determined using the force plate data (threshold of vertical ground reaction force: 20 N). The step angle, defined as the angle between the heel-to-toe vector and the global walking direction, was also determined at the moment of the next midstance. We subtracted the step angle on unperturbed steps from that on perturbed steps to obtain the step angle change. A positive step angle change means exorotation of the foot, while a negative one means endorotation.

The midpoint of toe and heel was used to describe the foot kinematics for the frontal and sagittal planes. Velocity was calculated using the central difference algorithm. Foot kinematic data were analysed without any filtering since they were smooth enough for latency calculation within each step. We defined the ‘response’ as the difference between perturbed steps and their references. The subtracted response isolates the effect of the target shift. We obtained the response latency by drawing a line through the points at which the response reached 25% and 75% of the peak response, and taking its intersection with baseline (Veerman et al. 2008). The slope of this line was defined as ‘vigour’ (acceleration). We used the average difference in velocity from 50 ms before to 50 ms after the (virtual) perturbation (which is very close to zero because this was the period used for matching the reference) as the baseline. We calculated the response latency for individual participants after averaging all their responses to a certain perturbation, except when determining the time dependency (see below). Latencies were determined for each leg and perturbation direction and subsequently averaged across legs.

Bodyweight shifts were evaluated from shifts in the COP as measured by the force plates. Kinetic data were filtered using a fourth-order low-pass Butterworth filter with a cutoff frequency of 20 Hz. Forces and moments were used to calculate the whole-body COP. The COP data were noisier than the kinematics, so we could not always determine the response in individual steps. However, enough data were available to make averages. We calculated the latency in the same way as for the foot velocity using the averaged response of each participant.

EMG data were first band-pass-filtered (20–400 Hz, fourth order), then rectified and filtered again through a low-pass filter (80 Hz, fourth order). Muscle activity (%) was normalised by individual MVC levels. Muscle activity on the selected unperturbed trials was subtracted from that on the perturbed trials to obtain the ‘response’ activity. The same extrapolation method was used to define the latency as for the other measures. Again, the latency was determined using the average response to a given perturbation for each participant. To be able to determine individual participants’ latencies for each perturbation, we had to decrease the low-pass cut-off frequency from 80 to 30 Hz, because otherwise some of the data were too noisy. EMG traces are shown both for the stance and the swing leg. The data of each reported muscle is based on at least 19 young participants and 15 older participants. (A few channels did not record properly during some measurements.)

As the exact timing of the target shift within the step cycle varied from step to step, we were able to check the time dependency of several kinematic variables. We did so for the extent of the correction, the peak response velocity and the response vigour. Time was expressed as the ‘remaining time’, which for a perturbed step was the time from the moment of onset of perturbation to heel-strike (also termed ‘available response time’ in the literature). The SMART method (van Leeuwen et al. 2019) was used to show the trend in the data points in a model-free manner. For this analysis, we can only use perturbed steps for ‘clear adjustments’ in which the response allows us to identify a peak, i.e. a peak foot velocity response of at least 5 cm/s between 50 and 330 ms after the target shift. We did not exclude any participants, but we removed perturbed steps without clear adjustments. In total we included 89% of the target shifts for the young and 77% for the older adults. The choice of the temporal resolution (standard deviation of the Gaussian kernel) had a negligible effect on the reconstructed time-course; we chose *σ* = 18 ms. We analysed the responses as a function of the remaining time for perturbations within the first half of the leg swing (from − 400 to − 200 ms). Since data from the two legs were similar, we combined the data of the left and right leg, and compared the differences between responses to medial and lateral perturbations using the SMART method.

### Statistics

Data are reported as mean ± standard deviation across participants. Latencies were determined for each perturbation direction and participant. Any participant’s latency outside 3 standard deviations from the mean for that perturbation direction was excluded as an outlier. A 2 × 2 Analysis of variance (ANOVA, age group: young/older, perturbation side: lateral/medial) was used to test whether these factors influenced correction, step angle change, and response latency of the foot and of the COP. As the unperturbed trials have their own duration, a 2 × 3 ANOVA was used for swing duration (age group: young/older, perturbation side: unperturbed/lateral/medial). Independent *t* tests were used to compare the TUG results and muscle response latencies between age groups. The relationship between age and the peak velocity of responses was tested using Pearson correlation. For all tests, *p* < 0.05 was considered as significant.

## Results

The older adults had an about 1.1 s longer TUG than the young adults (Table 1; the TUG test values of the young adults were not presented in the previous paper). All young adults responded clearly to the target shifts, as did most of the elderly. However, there were two older adults who corrected for less than 10% of the perturbation. One was 78 years old (the oldest), had the longest TUG (10.7 s) and corrected for less than 6% of the perturbation. The other was 72 years old, had a relatively fast TUG (6.7 s) and also corrected minimally. As shown in Table 1, and supported by the 2 × 2 ANOVA for correction, older adults corrected less than young adults (Young: 67%, Older: 40%). The medial correction was smaller than the lateral correction (*F*_(1,76)_ = 10.0, *p* = 0.002), with no significant interaction between age group and perturbation direction (*F*_(1,76)_ = 3.75, *p* = 0.057). Not surprisingly, for both age groups, the step response to the target shifts included some endorotation for medial perturbations and exorotation for lateral perturbations. The step angle changed towards the perturbation side, with larger amplitude (regardless of the sign) in older adults (Direction: *F*_(1,76)_ = 5.85, *p* = 0.018; Age × Direction: *F*_(1,76)_ = 0.34, *p* = 0.56).

**Table 1:** Comparison of Timed up and go (TUG), correction (corrected distance/2.5 cm × 100%), extent to which the step angle changes (positive is foot rotating outward), the duration of the swing phase of the movement (toe-off until heel-strike), the latency of the kinematic response and the latency of changes in centre of pressure (COP) in young and older adults

	Young	Older	Comparison
Age (years)	24.3 ± 3.6	70.0 ± 5.2	
*N*	20	20	
TUG (s)	6.3 ± 0.9	7.4 ± 1.1	*t*_(38)_ = 3.32, *p* = 0.002*
Correction (%)			
Lateral	76 ± 18	42 ± 18	F_(1,76)_ = 59.7, p < 0.001*
Medial	58 ± 12	37 ± 16	
Step angle change (deg)			
Lateral	1.0 ± 0.7	1.6 ± 0.9	*F*_(1,76)_ = 5.66, *p* = 0.020*
Medial	− 1.6 ± 0.9	− 2.0 ± 1.0	
Swing duration (ms)			
Unperturbed	397 ± 13	389 ± 13	*F*_(1,114)_ = 21.7, *p* < 0.001*
Lateral	410 ± 16	393 ± 16	
Medial	396 ± 15	385 ± 14	
Kinematic latency (ms)			
Lateral	154 ± 9	178 ± 19	*F*_(1,75)_ = 38.6, *p* < 0.001*
Medial	159 ± 11	180 ± 21	
COP latency (ms)			
Lateral	130 ± 20	143 ± 28	*F*_(1,72)_ = 8.07, *p* = 0.006*
Medial	144 ± 11	157 ± 16	

Age influenced all these measures significantly

The 2 × 3 ANOVA for swing duration showed that young adults had longer swing durations than older adults. There was also a significant effect of perturbation direction on swing duration (*F*_(2,114)_ = 6.03, *p* = 0.003). The post hoc LSD showed that the swing duration with lateral perturbations was 11 ms longer than that for medial perturbations (*p* = 0.001) and 8 ms longer than for unperturbed steps (*p* = 0.012). There was no significant difference between the swing durations of unperturbed and medially perturbed steps (*p* = 0.44). The perturbation occurred at around 28% of the swing duration in both groups.

**Fig. 2:**
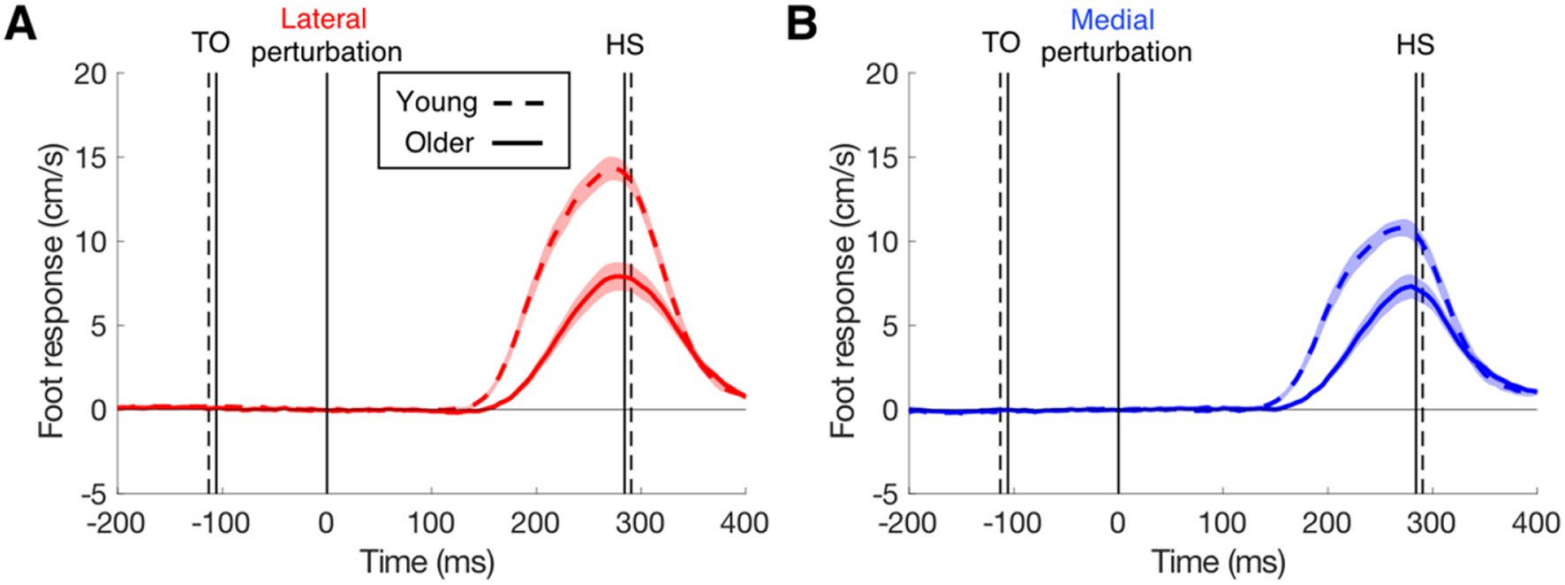
The change in medio-lateral velocity of the foot of the swing leg in the young and older participants as a function of the time after the perturbation. **a** Lateral target shifts. **b** Medial target shifts. Positive responses are in the same direction as the perturbation. Shaded areas represent the SEM across 20 participants. Vertical lines show the average onset (toe-off, TO) and offset (heel-strike, HS) of the swing leg (dashed for the young), along with the onset of the perturbation

Figure 2 shows the medio-lateral responses of the foot of the swing leg. The responses were all in the same direction as the perturbation, but differed in timing and amplitude between the two groups. The responses had 22 ms longer latencies in the older participants (Table 1). There was no effect of direction (medial or lateral; *F*_(1,75)_ = 1.02, *p* = 0.32) or interaction of direction with age (*F*_(1,75)_ = 0.16, *p* = 0.69). The response of the swing leg did not only result in a different final position, but also a different angle.

**Fig. 3:**
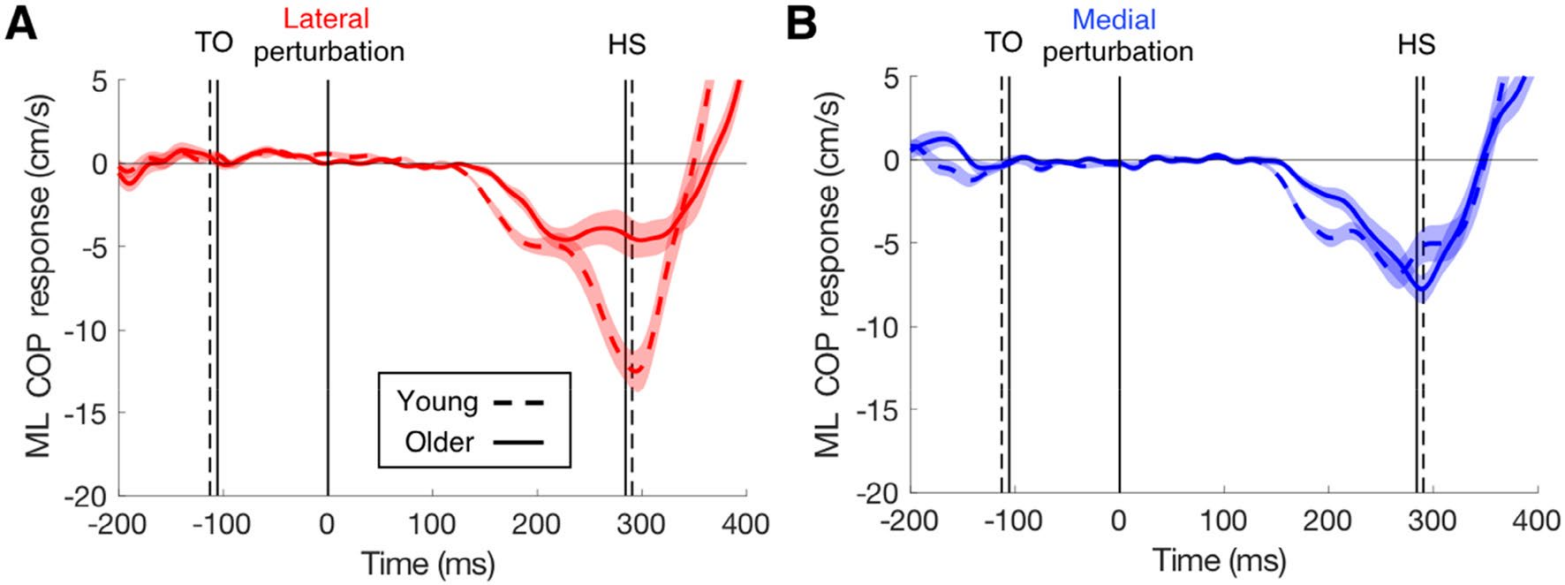
The medio-lateral COP responses in young and older participants to **a** lateral target shifts and **b** medial target shifts. Details as in Fig. 2

**Fig. 4:**
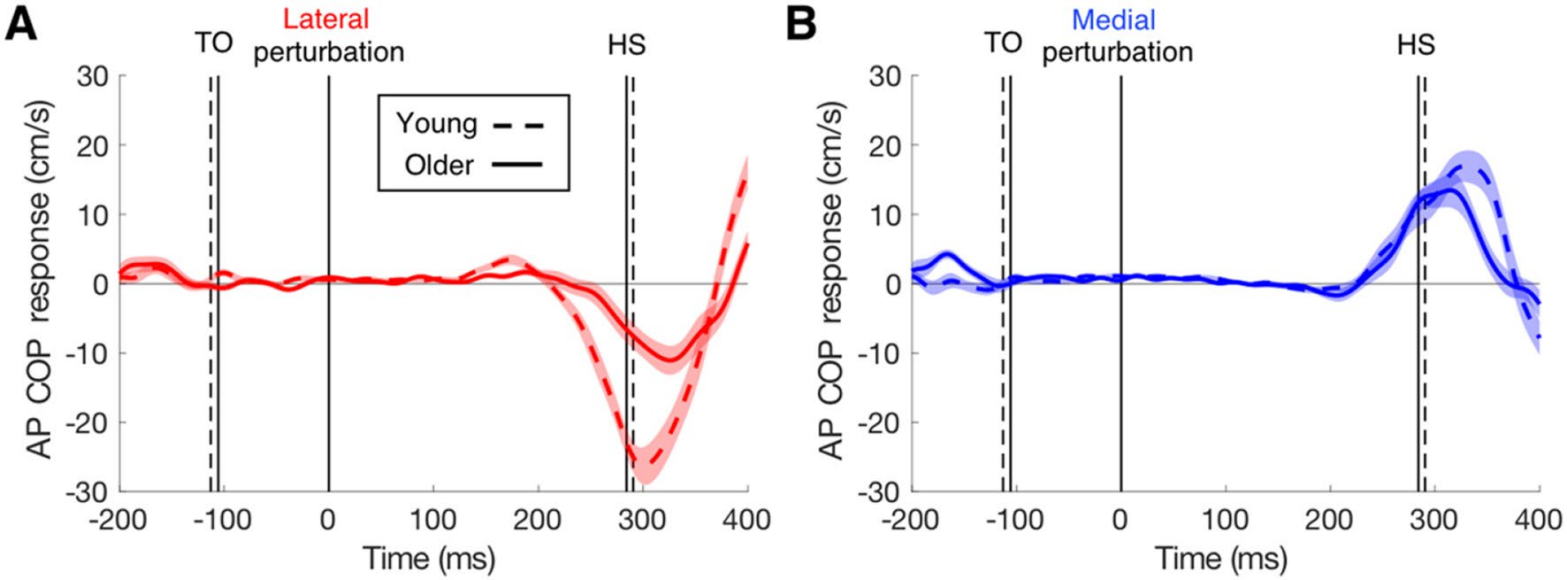
The anterior–posterior COP responses in young and older participants to **a** lateral target shifts and **b** medial target shifts. Details as in Fig. 2. Positive responses are in the anterior direction

The COP (under the stance leg) responded in the opposite direction than the foot of the swing leg (in the medio-lateral direction, Fig. 3). For instance, when a lateral target shift occurred during the right leg swing, the swing foot adjusted laterally and the COP under the left stance foot adjusted laterally. Hence, both responses were to the lateral side but in opposite directions. As was found for foot response latencies, age had an effect on COP response latencies. The latencies of the COP responses were 13 ms shorter in the young than in the older adults for both lateral and medial perturbations (Table 1). Medial responses had 14 ms longer latencies than lateral ones (*F*_(1,72)_ = 8.83, *p* = 0.004), and there was no interaction with age (*F*_(1,72)_ = 0.002, *p* = 0.97). COP also responded in the anterior–posterior direction, and did so in different ways for medial and lateral perturbations (Fig. 4). Specifically, the COP of the stance foot accelerated in the anterior direction for medial target shifts and decelerated in the anterior direction for lateral target shifts. Magnitude differences between COP responses of young and older adults were mainly evident for lateral target shifts, both for the medio-lateral direction (Fig. 3) and the anterior–posterior direction (Fig. 4).

**Fig. 5:**
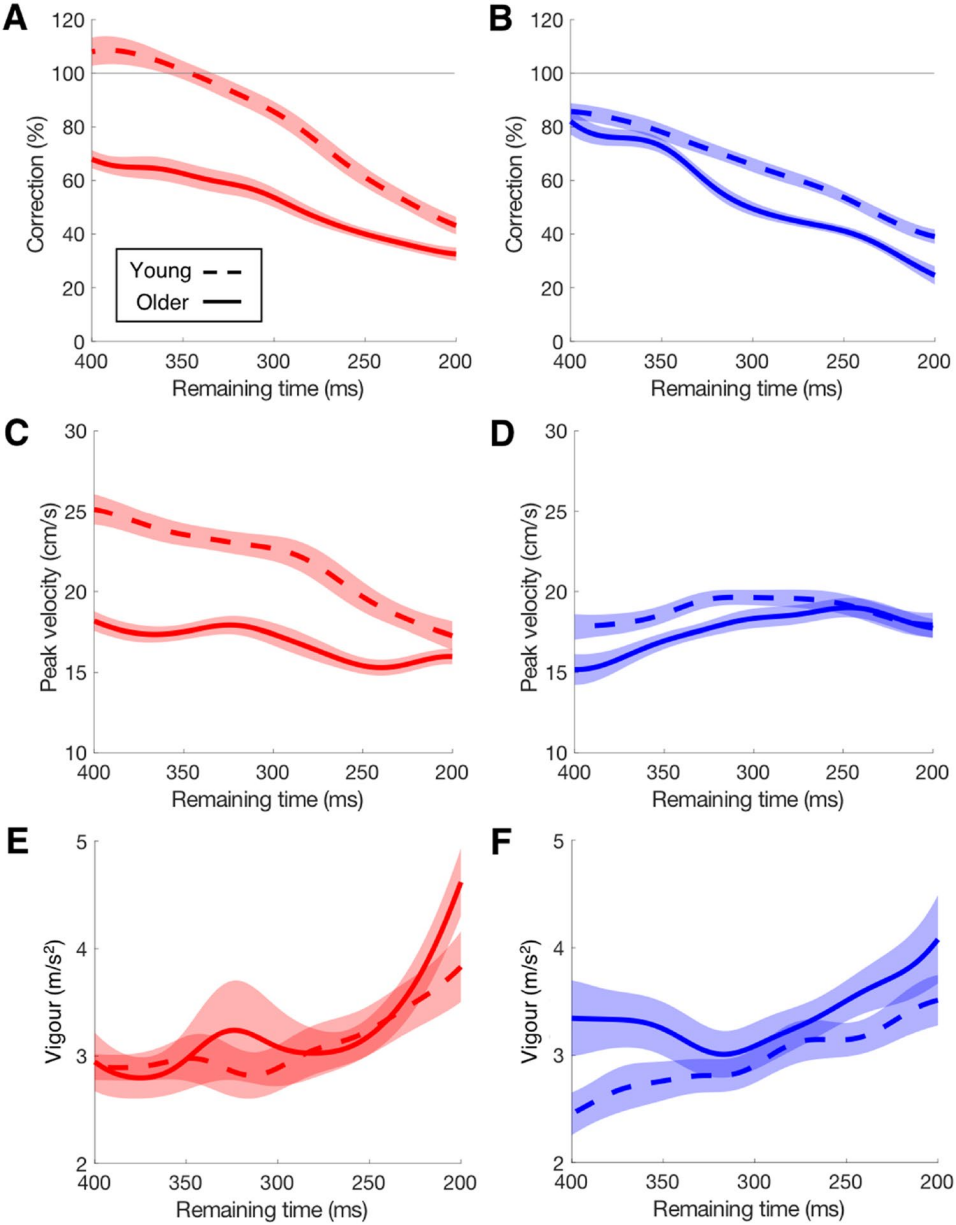
How features of the response to a lateral (red) or medial (blue) target shift depend on the timing of the perturbation (expressed as remaining time until heel-strike) in young and older participants. **a**, **b** Correction. Values larger than 100% mean that the foot is overshooting the target. **c**, **d** Peak velocity. **e**, **f** Vigour. Shaded areas represent the SEM across participants

**Fig. 6:**
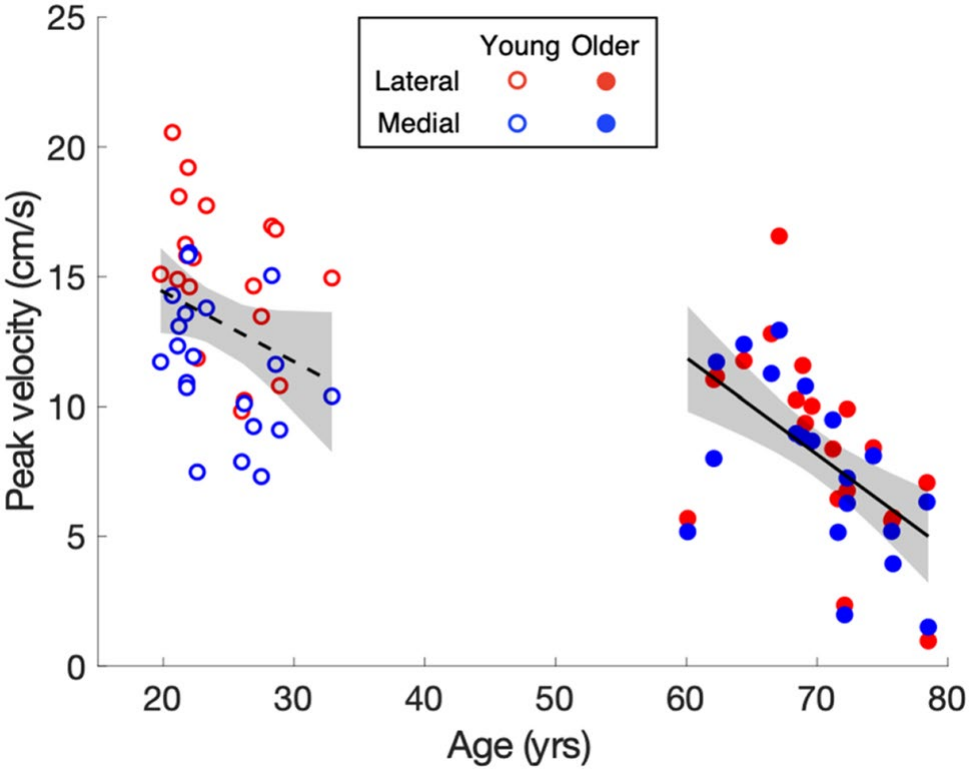
Relationship between age and peak velocity of the foot response to a lateral or medial target shift in young and older participants. Each dot represents the peak velocity of the mean foot response of one participant. The dashed and continuous lines are linear regressions calculated across directions for the young and older group, respectively. The slope with shaded area (95% confidence interval) in young adults is − 0.27 (− 0.56, 0.02), and in older adults − 0.37 (− 0.55, − 0.19)

Figure 5 shows the comparison of various characteristics of medio-lateral responses for the two age groups as a function of remaining time. As we need single-step estimates of the response for this analysis, we had to exclude steps for which the adjustment was small (for details see section ‘dependent variables’), so the resulting response values are higher than those in Fig. 2. The percentage correction decreased with the remaining time for both age groups (Fig. 5a, b). When only little time was available to adjust foot placement, correction of foot placement was similar between age groups for both lateral and medial adjustments. With more response time available (up to 400 ms), lateral corrections increased more in the young group (Fig. 5a, b). A similar pattern was found in peak response velocity (Fig. 5c, d). When a perturbation occurred later in the swing phase, just before the heel-strike, the responses were more vigorous (larger acceleration), regardless of age or perturbation direction (Fig. 5e, f). Looking at the relationship between age and peak response velocity in more detail (Fig. 6), we see a significant negative correlation between peak response velocity and age for the older group (*r* = − 0.56, *p* < 0.001), but not for the young group (*r* = − 0.29, *p* = 0.067). The reduced correlation in the young group could just be due to the narrower age-range for the young participants, but it is likely that the overall relationship is quadratic rather than linear (Lexell et al. 1988), so that the decline in velocity is stronger in the elderly. A similar age dependency exists for the ability to avoid obstacles: the success rate declines with age (Weerdesteyn et al. 2005).

**Fig. 7:**
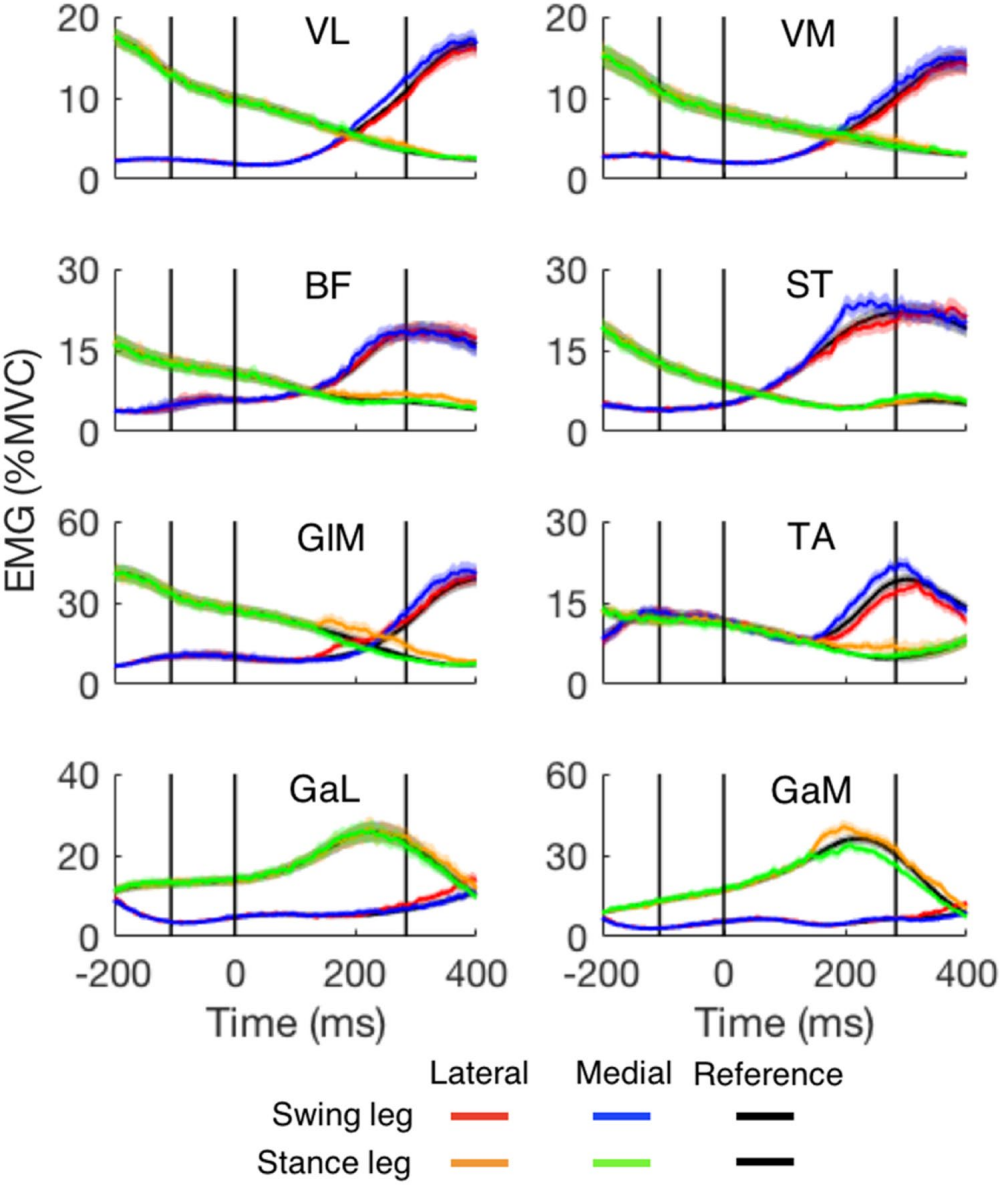
The EMG of all eight muscles of both the swing leg and stance leg for steps with medial and lateral target shifts as a function of time after the target shift (older participants only). The EMG in the corresponding reference steps is indicated by the black traces. Vertical lines from the left to right show the average moments of toe-off, perturbation and heel-strike. For each muscle, at least 15 participants are included. *VL* vastus lateralis, *VM* vastus medialis, *BF* biceps femoris, *ST* semitendinosus, *GlM* gluteus medius, *TA* tibialis anterior, *GaL* gastrocnemius lateralis, *GaM* gastrocnemius medialis. Note that the scales differ from those of a similar plot for the young adults in our previous paper (Fig. 6 in Zhang et al. (2020))

The muscle activation patterns during unperturbed stepping for the elderly (black curves in Fig. 7) show a normal activation pattern. The main difference with the young adults (not shown; for their patterns see Fig. 6 in Zhang et al. 2020) is that all muscles in the older adults were more active. This might reflect the reduced muscle strength in the elderly, because activity was expressed in relation to that when exerting maximal force. The responses to target shifts are less clear for the elderly.

**Fig. 8:**
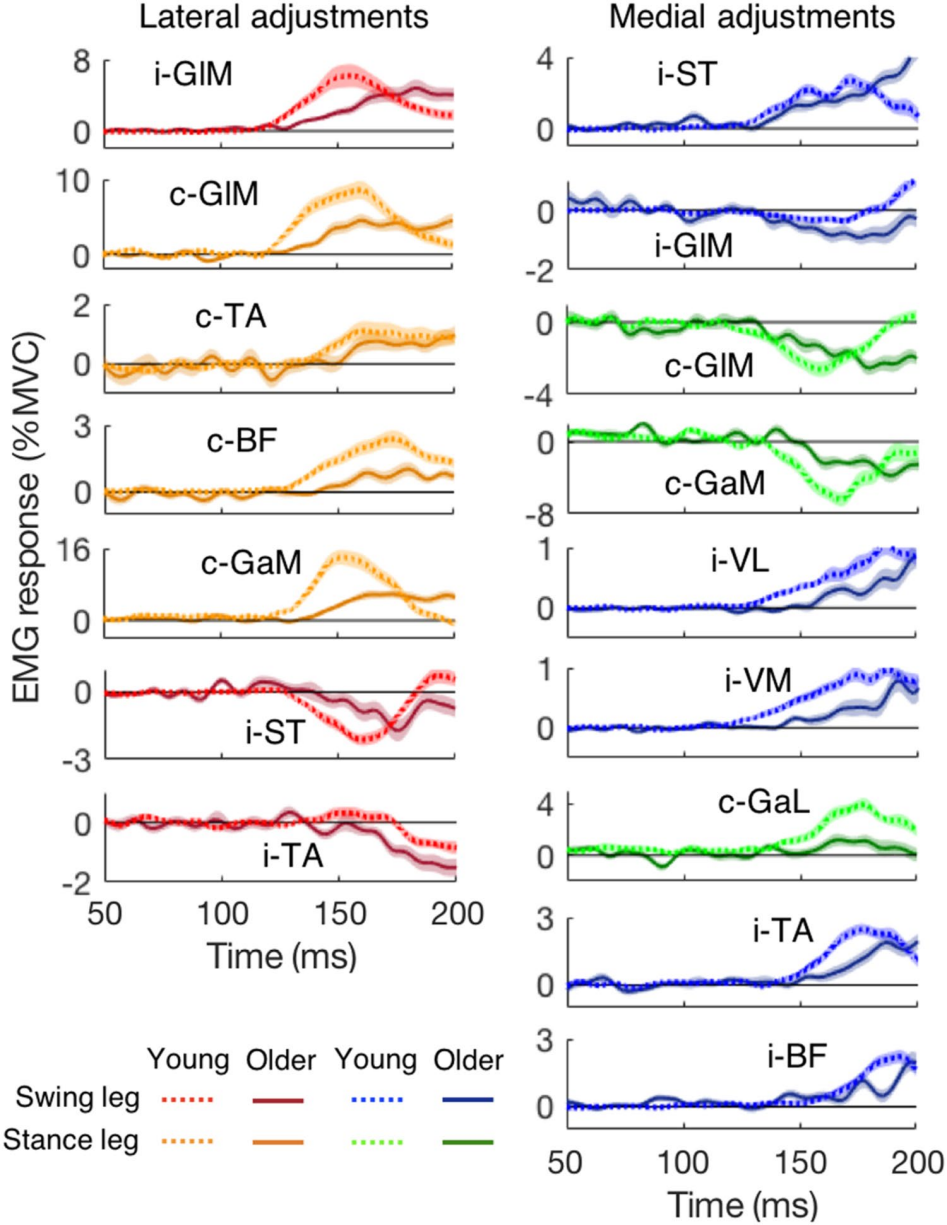
Comparison of main muscle activity changes in response to target shifts as a function of the time after the perturbation, averaged across all target shifts and all participants in each age group in a short-to-long latency order. Note that, due to the way the figure was constructed, the latency of the responses may seem to differ slightly from the overall latency of all participants depicted in Table 2. Left column: lateral target shift. Right column: medial target shift. The ‘i-’ and ‘c-’ in front of the muscle names indicate ipsilateral (the swing leg) and contralateral (the stance leg), respectively. Muscle abbreviations are detailed in the caption of Fig. 7

Though muscles in the older adults were more active than those in the young adults during unperturbed steps (expressed in % MVC), their activity changes in response to target shifts were generally smaller than those of the young. This is for instance the case for c-GlM, c-BF and c-GaM for lateral shifts, and c-GaM and c-GaL for medial shifts (Fig. 8). However, two muscles showed larger magnitude of responses in the older adults than in the young adults: i-TA for lateral shifts and i-GlM for medial shifts. Other differences were relatively minor. The similarities between the responses in young and older adults were much more striking than the differences. In general, the responding muscles were recruited in the same order. For example, just as in young adults, the earliest muscles to respond were i-GlM and c-GlM for lateral perturbations, and i-ST and c-GlM for medial perturbations. For most muscles, the latency was slightly longer in the older adults. For instance, for lateral shifts, the early-responding muscles (i-GlM and c-GlM) were delayed by about 10 ms in older adults. There were some exceptions. Some muscle responses (i-ST and c-GlM) for medial shifts were not delayed in the older adults (Table 2). For one muscle (i-GlM), the latency of the suppression for medial shifts was shorter in the older adults.

**Table 2:** Response latencies to lateral and medial target shifts for early responding muscles. The number of participants for each muscle is presented in brackets

	Young	Older	Comparison
Lateral shifts (ms)			
i-GlM	122 ± 9 (20)	133 ± 20 (19)	*t*_(23.9)_ = 2.3, *p* = 0.031*
c-GlM	123 ± 7 (20)	131 ± 17 (19)	*t*_(23.7)_ = 2.1, *p* = 0.047*
Medial shifts (ms)			
c-GlM	128 ± 19 (20)	131 ± 29 (19)	*t*_(37)_ = 0.4, *p* = 0.663
i-ST	141 ± 19 (20)	136 ± 28 (19)	*t*_(37)_ = 0.7, *p* = 0.464
i-GlM	168 ± 23 (19)	140 ± 32 (19)	*t*_(36)_ = 3.1, *p* = 0.004*

## Discussion

This study investigated the effects of ageing on online adjustments to changes in target position when walking. We found that older adults could make such adjustments, but not as well as the young, in agreement with previous work (Mazaheri et al. 2015). This is a similar pattern to the one we had previously found for responses of the hand to target shifts (Zhang et al. 2018a). Ageing affected the EMG responses much less than the kinematics, suggesting that it is mainly the execution of the command that is affected, rather than the neural control.

### Medial versus lateral adjustments

The present data show that ageing had large effects on lateral adjustments, but much smaller effects on medial adjustments. Older adults tended to correct for less of the target shift than young adults (Table 1). The interaction between age and perturbation direction was not significant (*p* = 0.057). When the magnitude of the correction was related to the remaining time (Fig. 5a, b) there were still large age-related differences in responses to lateral target shifts, but not to medial shifts. A similar pattern was visible for the effect of ageing on the COP responses to perturbations in the two directions (Figs. 3, 4). The older adults had a reduced COP response amplitude for perturbations in the lateral direction, but similar amplitude for perturbations in the medial direction.

Muscles responded in the same order irrespective of age, with the exception of i-GlM that responded earlier in the elderly. The EMG activity of the older adults responded later than that of young adults to lateral target shifts. For medial target shifts, this difference was absent. The older adults even suppressed i-GlM faster and more strongly than the young in response to medial target shifts (Table 2 and Fig. 8). This suppression may be an attempt to reduce abduction for medial adjustments. Another muscle with stronger and earlier suppression in the older adults was i-TA for lateral target shifts (Fig. 8). A stronger suppression of i-TA in the elderly could contribute to their larger step angle change (Table 1). Sun et al. (2017) found similar results of the step angle change: older adults had more exorotation to lateral target shifts and more endorotation to medial target shifts.

The reason for smaller medial than lateral adjustments for both young and older adults could be that adjustments of the foot in the medial direction are particularly challenging as these require narrowing the margin of balance (Moraes et al. 2007). In that case, the requirement to ‘catch’ the body limit the adjustment of the foot (Bancroft and Day 2016). Indeed, when stepping on adaptable targets, stepping errors are reduced when balance is supported by crutches (van der Veen et al. 2020). Lateral adjustments lead to an increase in the base of support, while medial adjustments lead to a decrease. This balance challenge is also reflected in other studies. It has been demonstrated that the centre of mass (COM) trajectory can be adjusted depending on the intended foot placement direction, which is in the same direction for lateral foot adjustments but not much for medial adjustments (Lyon and Day 1997, 2005). The limitation in medial adjustments is more profound in stroke patients who have balance difficulties (Nonnekes et al. 2010). Understandably, participants try to avoid destabilising adjustments. This might explain why medial adjustments are difficult, especially when the perturbation sizes are large (Hoogkamer et al. 2015; Moraes et al. 2007).

### EMG versus kinematics

Perhaps the most striking result of the present study was that the delay with ageing was larger in the behaviour (kinematics) than in the EMG. The delay of the foot responses was around 22 ms (Fig. 2). In contrast, the delay in muscle responses was only about 10 ms (lateral adjustments in Table 2). Moreover, for medial adjustments, some muscles in the older adults showed no delay (i-ST and c-GlM). These results indicate that the deficit in corrections is not only due to the underlying deterioration of the central processing, but to a large extent due to a failure to quickly translate the changes in motor unit activation into changes in muscle force. In line with this, we found a clear decrease in peak velocity with age, especially for the older adults (Fig. 6).

A further argument for the preservation of basic neural control in the older participants was that most muscles responded in the same order as they did in the young. The functions of those muscles in step adjustments have been explained in our previous study (Zhang et al. 2020). However, in general, the older adults had slightly (< 10 ms) later responses. Combined with weaker muscles, this could result in later and smaller adjustments of the COP and of the swing foot, and this could lead to less correction.

### Arm reaching versus foot stepping

It has been argued that there are many similarities in the neural control of upper-limb and lower-limb reaching (Drew and Marigold 2015; Georgopoulos and Grillner 1989; Yakovenko and Drew 2015; Yakovenko et al. 2011; Zhang et al. 2020). The present findings on fast adjustments to shifting targets are in line with these ideas. An age-related delay of 22 ms for the kinematic response of the foot (Table 1) is fully comparable to the reported delays (15–20 ms) in arm reaching tasks with target displacements (Kadota and Gomi 2010; Kimura et al. 2015; Zhang et al. 2018a). Even with these consistently longer delays for the older participants, the present adjustments were fast enough to qualify as online corrections. Hence, it is fair to assume that online corrections are preserved in older adults. However, delays in the execution of these corrections may still increase the risk of falls in this population.

Some differences between arm and leg reaching were observed. The fundamental difference between upper and lower limb tasks is that losing balance is generally not an issue in arm reaching (Voudouris et al. 2013), but quite an issue in walking. The balance issue also affects the direction of the medio-lateral adjustments. In arm reaching the direction of the perturbation has little or no effect on the adjustments (Oostwoud Wijdenes et al. 2013). In contrast, in the present study it was found that corrections for lateral shifts were more complete than those for medial shifts. Another difference is that step adjustments must be made within the limited time that is available before the heel-strike, hence with more time pressure than in arm reaching. In steady walking on a treadmill with fixed speed, both young and older adults could hardly increase their limited time in a step to complete insufficient corrections. The present results show that older adults did not prolong their swing duration (they even shortened it slightly, Table 1), and the accuracy decreased substantially when the remaining time (between target shift and heel-strike) decreased (Fig. 5a, b). However, for arm reaching the whole movement time could be prolonged. Our previous work showed that older adults responded about 15 ms later than young adults, with their whole movement time prolonged by about 160 ms (Zhang et al. 2018a). They probably increased the movement time to keep their movements equally accurate as in the young.

There are some limitations of the present study. While sampling rate for EMG was high, the sampling of the video (240 Hz) and Vicon (200 Hz) limited the available measurement resolution. On the other hand, this resolution was in line with most of the studies in this field.

## Conclusions

Our study suggests that older adults can adjust their steps to small target shifts during the swing phase, but with delayed responses and with a smaller correction than young adults. The prolongation in response latency was similar to that in reaching. Ageing delayed the behavioural responses more than it did the EMG responses, suggesting that reduced muscle strength is partly responsible for the changes in online adjustments during ageing. It remains for future work to establish whether training can compensate for this and lead to improved performance.

## Data Availability

The datasets generated during and/or analysed during the current study are not publicly available due to the privacy protection (Belgian Act of 8 December 1992 on the protection of privacy and the Belgian Act of 22 August 2002 on patient rights, which were mentioned in the informed consent).

## Abbreviations

ANOVA: Analysis of variance
BF: Biceps femoris
c-: Contralateral
COP: Centre of pressure
EMG: Electromyography
GaL: Gastrocnemius lateralis
GaM: Gastrocnemius medialis
GlM: Gluteus medius
i-: Ipsilateral
MVC: Maximal voluntary contraction
SEM: Standard error of the mean
ST: Semitendinosus
TA: Tibialis anterior
TUG: Timed up and go
VL: Vastus lateralis
VM: Vastus medialis

## Code availability

The properties of stepping-targets and their order of appearance were coded in MATLAB (The MathWorks Inc, USA) and loaded to CueFors (Motekforce Link, the Netherlands). Kinematic, kinetic and muscle activity data were analysed in MATLAB.
